# Optic Flow Simulating Self-Motion Does Not Modulate the Hoffmann Reflex in the Soleus During Upright Standing in Healthy Young Adults

**DOI:** 10.3390/brainsci16030297

**Published:** 2026-03-06

**Authors:** Christophe Barbanchon, Stéphane Baudry

**Affiliations:** NeuroMove—Laboratory of Movement Neurophysiology, Faculty of human Movement Sciences, Université Libre de Bruxelles, 1070 Bruxelles, Belgium; christophe.barbanchon@ulb.be

**Keywords:** virtual reality, postural control, sensorimotor integration, sensory re-weighting

## Abstract

**Highlights:**

**What are the main findings?**

- Optic-flow stimulation increases postural sway but does not induce modulation of the soleus Hoffmann reflex, regardless of optic-flow complexity.
- Supported-standing trials confirm that optic flow alone (without postural engagement) fails to alter segmental reflex.

**What are the implications of the main findings?**

- Optic flow alone is insufficient to alter Ia-monosynaptic reflex gain in non-threatening contexts.
- Postural control in response to virtual optic flow could mainly depend on supraspinal mechanisms under non-threatening conditions.

**Abstract:**

**Background/Objectives**: Visual motion is a powerful contributor to postural control, yet its influence on modulation of the Ia afferent pathway remains to be confirmed. This study investigated whether optic-flow simulating self-motion modulates the soleus Hoffmann (H) reflex recorded in the soleus during upright stance in immersive virtual reality. **Methods**: Fourteen healthy adults completed two experimental sessions, each comprising four visual conditions of increasing optic-flow complexity. In one session, participants stood freely on a force platform (free standing) whereas in the other, postural sways were restricted (supported standing). Surface EMG, posterior tibial nerve stimulation, and force-platform recordings were collected. **Results**: During free standing, optic flow substantially increased postural sway [F(3,13) = 15.7, *p* < 0.001, η^2^ = 0.55], with higher sway in all optic-flow conditions (~13 mm/s) compared with static viewing (~10 mm/s). In contrast, soleus H-reflex amplitude was not modulated by optic flow [F(3,13) = 0.2, *p* = 0.57], remaining stable across conditions (~44% M_max_). Background EMG and CoP position preceding stimulation were similar across conditions. In supported standing, used to isolate the effect of optic flow independently to postural control, H-reflex amplitude again showed no condition effect [F(3,13) = 0.2, *p* = 0.86]. **Conclusions**: These findings indicate that postural perturbation induced by optic flow was not accompanied by a modulation of the Ia afferent-motoneuron transmission of the soleus under the used experimental conditions. The results suggest that postural control under virtual optic flow is mediated predominantly by supraspinal sensory-integration mechanisms, rather than by modulation of the Ia-monosynaptic reflex pathway.

## 1. Introduction

Maintaining upright stance relies on the continuous integration of information from visual, vestibular, and proprioceptive systems to stabilize the body against internal and external perturbations [1]. Optic flow provides powerful information about self-motion and environmental stability. Classical “moving-room” paradigms demonstrated that even small shifts in visual surroundings produce robust postural responses coherent with the direction of optic flow, indicating that the visual system heavily constrains postural control [2,3]. Modern virtual reality (VR) setups confirmed these findings, further showing that visually simulated self-motion elicits consistent body sway [4,5].

Exposure to optic flow alters the upright standing stability by disrupting the sensorimotor integration through sensory conflict between visual, vestibular, and proprioceptive cues [1,6]. VR optic flow simulating forward and backward self-motion are highly effective in eliciting vection and robust postural adjustments [4,7,8]. Typically, forward optic flow induces perceived forward displacement and triggers backward compensatory sway, whereas backward optic flow evokes forward sway. Importantly, these visually driven postural reactions emerge even in the absence of physical perturbations, supporting vision as a powerful contributor to the internal estimate of body orientation [2,4,5].

While such studies have focused primarily on behavioral balance responses, research examining whether such responses are accompanied by specific spinal neural adjustments to control leg muscles remains limited, although a large body of the literature reported specific spinal modulation depending on postural task demand [9,10,11]. Recent work reported that VR environments can modulate the efficacy of Ia afferents to discharge motoneurons, assessed with the Hoffmann (H) reflex [12]. In their study using “virtually simulated falling,” these authors showed that exposure to a visually threatening virtual scenario decreased the H-reflex amplitude of the soleus (SOL) muscle, despite minimal changes in muscle activity or body oscillations. Their findings indicate that visual context alone can adjust spinal reflex gain through top-down mechanisms. In another study, VR height exposure increased arousal and postural sway while simultaneously decreasing H-reflex amplitude, implicating supraspinal mechanisms sensitive to psychological state and environmental threat [13]. The above studies, however, did not investigate the effect of optic flow simulating self-motion, known to perturb postural control. Immersive VR environments are known to recruit cortical and subcortical structures involved in spatial orientation, sensory integration, and postural threat appraisal [14,15], providing plausible supraspinal pathways through which optic flow may influence spinal-level processing. 

The present study aimed to determine whether the amplitude of the soleus H-reflex is modulated during upright standing in an immersive VR environment simulating self-motion. Based on previous work showing that H-reflex amplitude decreases with balance task difficulty [9,10,11] and the presence of balance threat [12,16], we hypothesized that optic flow would depress soleus H-reflex amplitude, reflecting the influence of optic flow on the spinal reflex pathway. As optic flow alters perceived postural stability without changing actual biomechanics, H-reflex modulation would occur independently of changes in background EMG, consistent with the hypothesis that visual inputs bias the central set regulating reflex gain during upright standing.

## 2. Methods

### 2.1. Participants

An a priori sample size calculation was performed using G*Power software (version 3.1), assuming a large effect size [12], an alpha level of 0.05, and a power of 0.80, which indicated a required total sample size of 10 participants. We recruited additional participants as a conservative approach to face potential dropout or technical issues. Sixteen adults [mean (SD); age: 22.7 (2.5) years; 3 women] volunteered to take part in this study after giving their written informed consent. Participants were enrolled in the study if they did not have neurological disorders with potential residual motor deficits (stroke, Parkinson disease, multiple sclerosis, etc.), diabetes, epilepsy, cardiac history, orthopedic issues involving the lower limbs, and did not take medications that could influence balance (sedatives, hypnotics, antidepressants, and benzodiazepines). Approval for the project was obtained from the local Ethics Committee, and all procedures used in this study conformed to the Declaration of Helsinki.

### 2.2. Surface Electromyogram

Delsys Trigno wireless instrument (Delsys Inc., Natick, MA, USA) was used for recording surface electromyography (EMG) from SOL, gastrocnemius medialis (GM) and tibialis anterior (TA) muscles from the non-dominant leg. The dominant leg was determined by asking an individual to kick a ball [17]. Before attaching the sensors, skin was shaved when necessary and cleaned with a solution of alcohol, ether, and acetone to reduce the impedance at the skin–electrode interface. The sensor for SOL was placed 2 cm below the muscle–tendon junction of the GM in line with the Achilles tendon. The sensor for the GM was placed midway between the femoral condyle and the muscle–tendon junction. The sensor for the TA was placed at one-third of the distance between the fibular head and the lateral malleolus and 1 cm lateral to the tibia. Surface EMG signals were recorded at a sampling rate of 1926 Hz. The sensor features an analog bandpass (20–450 Hz) with 2nd-order high-pass and 4th-order low-pass filters. The wireless multi-channel EMG signal was A/D converted (Power 1401, 16-bit resolution, Cambridge Electronic Design, Cambridge, UK) before being stored on a computer for subsequent analyses.

### 2.3. Peripheral Nerve Stimulation

Electrical stimuli (1 ms duration) were delivered via a constant current stimulator (DS7A, Digitimer, Letchworth Garden City, UK) connected to cup electrodes (Ag-AgCl electrodes, 8 mm diameter), filled with gel, and attached to the skin of the non-dominant leg. The anode was located above the patella and cathode in the popliteal fossa. The optimal position of the cathode was defined as the location where the stimulation of the posterior tibial nerve elicited the largest H-reflex amplitude in the SOL at a given intensity of stimulation. During the experiment, stimulation intensity was selected to evoke a H reflex in the ascending phase of its recruitment curve preceded by an M wave with an amplitude comprised between 5 and 10% of the maximal M-wave amplitude (M_max_) [18], while the M_max_ was elicited using an intensity of 1.2 times the minimum current required to reach M_max_. The intensity associated with the H reflex and M_max_ were determined by progressively increasing the stimulation intensity by steps of 0.5–1 mA and 3–5 mA, respectively. Three responses for each intensity were evoked for each step.

### 2.4. Force Platform

A force platform (OR6-6-2000, Advanced Mechanical Technology, Watertown, MA, USA) was used to compute posturography variables. The signals from the platform were sampled at 100 Hz, A/D converted (Power 1401, 16-bit resolution, Cambridge Electronic Design, UK) and stored on a computer.

### 2.5. Virtual Reality

The “Optic Flow” program, included in the “Balance VR pack” developed by Virtualis (website: www.virtualisvr.com), was used, which consists of placing the participant in a virtual environment representing a tunnel, but with no end in sight. The virtual environment was displayed through a headset Vivo-Pro, which is equipped with G-sensor (2880 × 2800 combined pixels; 1440 × 1600 per eye; AMOLED). In all experimental conditions, participants were instructed to stand on the force platform in a bipedal position with a 10 cm distance between heels and the forefeet oriented laterally with a 30-degree angle between them (each foot rotated 15° from the sagittal plan). Participants kept their arms at their sides and were instructed to refrain from any head or limb movements.

Four conditions with different optic flow complexities were used to induce different levels of sensory demand that may require modulating the H-reflex pathway. Each condition lasted 90 s and was repeated four times, with three trials during which H reflexes and M_max_ were evoked (see below). Between each trial, the participant sat down for approximately 90 s. A first condition (A) consisted of being immersed in the VR environment without any optic flow simulating self-motion. A second condition (B) included alternating optic flow simulating forward and backward self-motion. A third condition (C) consisted of alternating optic flow simulating forward and backward self-motion combined with lateral directions (forward-left or forward-right) and (backward-left or backward-right). The last condition (D) was similar to C with the addition to clockwise and counterclockwise rotations of the visual field. The order of the scenarios was determined randomly using a computer program.

### 2.6. Experimental Protocol

Each participant performed two experimental sessions during which the four optic flow conditions were performed either without support (free standing; one session) or with support (supported standing; one session). The order of the sessions was randomized across participants with one week between the two sessions that were scheduled at the same time of the day. The objective of the supported standing session was to assess the influence of optic flow on afferent inputs at the spinal level by disentangling the direct effects of optic flow from those arising through postural adjustments. During the supported upright standing, participants stood upright and were strapped to an adjustable board placed in front of them at chest level [19].

**Figure 1:**
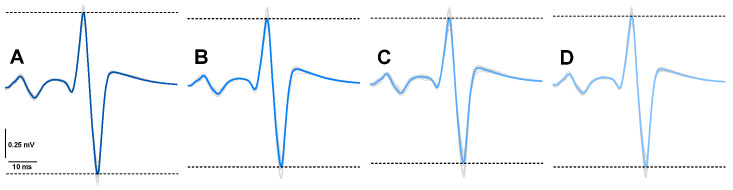
Original traces of the Hoffmann reflex and the preceding M wave for one participant in the four optic flow conditions: (**A**): no optic flow simulating self-motion; (**B**): alternating optic flow simulating forward and backward self-motion; (**C**): alternating optic flow simulated forward and backward self-motion combined with lateral directions (forward-left or forward-right) and (backward-left or backward-right). (**D**): similar to (**C**) with the addition of clockwise and counterclockwise rotations in the visual field. The gray traces represent the individual recordings while the color one illustrates the average trace.

During each trial, five H reflexes were triggered with a random interstimulus interval of 10–15 s to avoid participant anticipation. The first stimulation was triggered 10 s after the start of the trial. A stimulation to evoke an M_max_ was triggered after the 5 H reflexes (Figure 1).

### 2.7. Data Reduction

The displacement of the CoP was analyzed offline using Spike2 software (Cambridge Electronic Design, UK) with specific command lines. Initially, force platform signals underwent low-pass filtering (cut-off frequency: 10 Hz) using a fourth-order Butterworth filter. Subsequently, from the filtered data, the mean velocity of center of pressure (CoP) displacement in anterior–posterior (AP) and medio-lateral (ML) axes, and both axes together, was calculated for a 80 s period avoiding the first 10 s during the trial without peripheral nerve stimulation. The mean velocity of the CoP was chosen as it is considered one of the most reliable CoP measures [20] and to be predictive of balance capacity [21]. During trials with peripheral nerve stimulation, the rectified average value of the EMG for SOL, GM and TA was measured during the 100 ms epoch that preceded the stimulation to provide an index of the background muscle activity at the time of the stimulation. During the same period, the mean position of the CoP in the anterior–posterior axis was measured to provide information on the possible influence of postural sway on H-reflex amplitude [10].

### 2.8. Statistical Analysis

All statistical analyses were performed using the JASP software (version 00.19.3). Two participants did not come back to the lab for the second experimental session (one for the *free standing* condition and the other for the *supported standing* condition). Therefore, statistics were performed on 14 participants. After the Gaussian distribution was confirmed using a Shapiro–Wilk test for each dependent variable (soleus H-reflex amplitude, M-wave amplitude, pre-stimulus EMG activity of the SOL, GM and TA, and CoP position and velocity), a one-way repeated-measures ANOVA was conducted with conditions (A, B, C, D) as the within-subjects factor. When the assumption of sphericity was violated, as indicated by Mauchly’s test, Greenhouse–Geisser corrections were applied. Significant main effects were followed by Holm-corrected pairwise comparisons. Effect sizes are reported as partial eta squared (η^2^_p_). In addition, correlations between H-reflex amplitudes and CoP anterior–posterior displacement or pre-stimulus EMG levels were examined using Pearson’s coefficient. Values are presented as mean (standard deviation). Statistical significance was set at *p* < 0.05 for all analyses.

## 3. Results

### 3.1. Free Standing

#### 3.1.1. CoP Velocity

**Figure 2:**
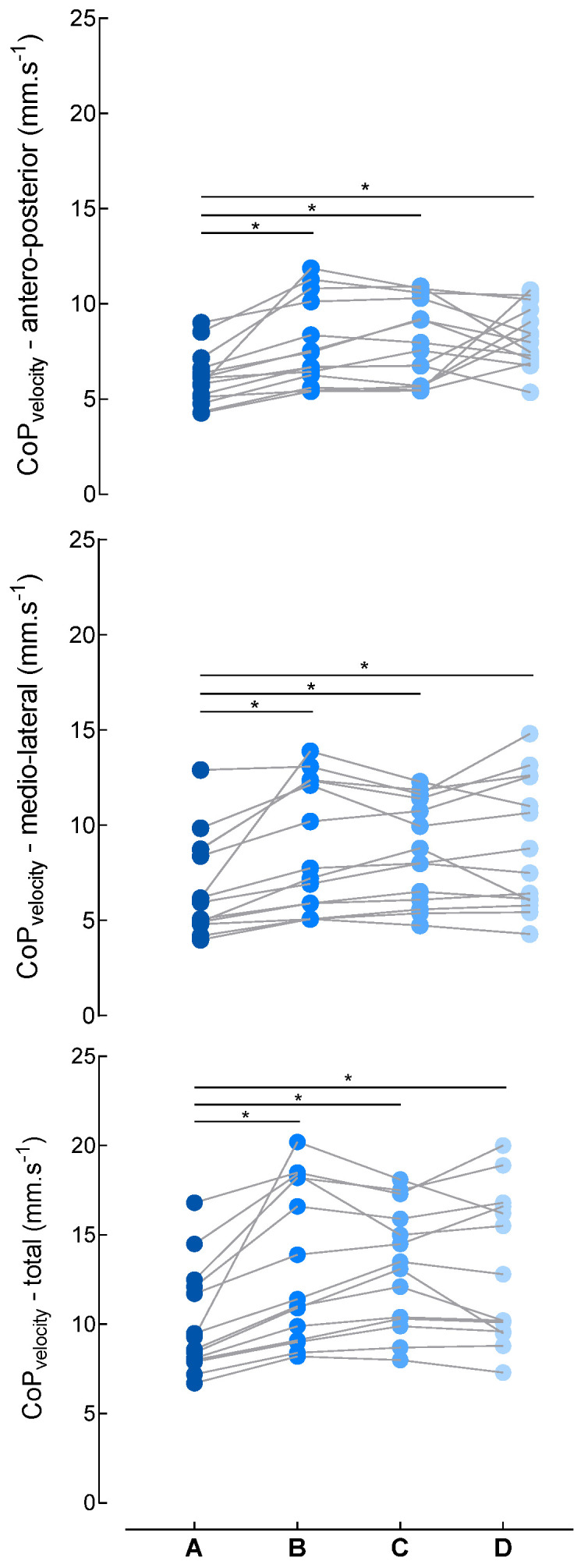
Mean velocity of the center of pressure (CoP_velocity_) for each condition of optic flow (A, B, C, D) in the antero-posterior (**top panel**) and medio-lateral axes (**mid panel**), and in both axes combined (**bottom panel**). A: no optic flow simulating self-motion; B: alternating optic flow simulating forward and backward self-motion; C: alternating optic flow simulated forward and backward self-motion combined with lateral directions (forward-left or forward-right) and (backward-left or backward-right). D: similar to C with the addition to clockwise and counterclockwise rotations in the visual field. Each point represents one participant. * denotes significant difference with condition A (*p* > 0.01).

CoP velocity in the antero-posterior axis demonstrated a strong and significant effect of conditions [F(3,13) = 7.4, *p* < 0.001, η^2^ = 0.379], with post hoc comparisons revealing that absence of simulated self-motion (condition A) was associated with substantially lower CoP velocity than all optic-flow conditions (B, C, D) (Figure 2, top panel). Specifically, CoP velocity in condition A was significantly lower than in conditions B (*p* = 0.004), C (*p* < 0.001), and D (*p* = 0.008), whereas no significant differences were observed between conditions B, C, and D (all *p* values > 0.05). Similarly, CoP velocity in the medio-lateral axis demonstrated a strong and significant effect of conditions [F(3,13) = 11.7, *p* < 0.001, η^2^ = 0.475], with post hoc comparisons revealing that absence of simulated self-motion (condition A) was associated with substantially lower CoP velocity than all optic-flow conditions (B, C, D) (Figure 2). Specifically, CoP velocity in condition A was significantly lower than in conditions B (*p* = 0.009), C (*p* = 0.009), and D (*p* < 0.001), whereas no significant difference was observed between conditions B, C, and D (all *p* values > 0.05). Finally, CoP velocity, when combining the two axes, demonstrated a strong and significant effect of conditions [F(3,13) = 15.7, *p* < 0.001, η^2^ = 0.547], with post hoc comparisons revealing that absence of simulated self-motion (condition A) was associated with substantially lower CoP velocity than all optic-flow conditions (B, C, D) (Figure 2). Specifically, CoP velocity in condition A was significantly lower than in conditions B (*p* = 0.004), C (*p* < 0.001), and D (*p* < 0.001), whereas no significant differences were observed between conditions B, C, and D (all *p* values > 0.05).

#### 3.1.2. H Reflex and M Wave

**Figure 3:**
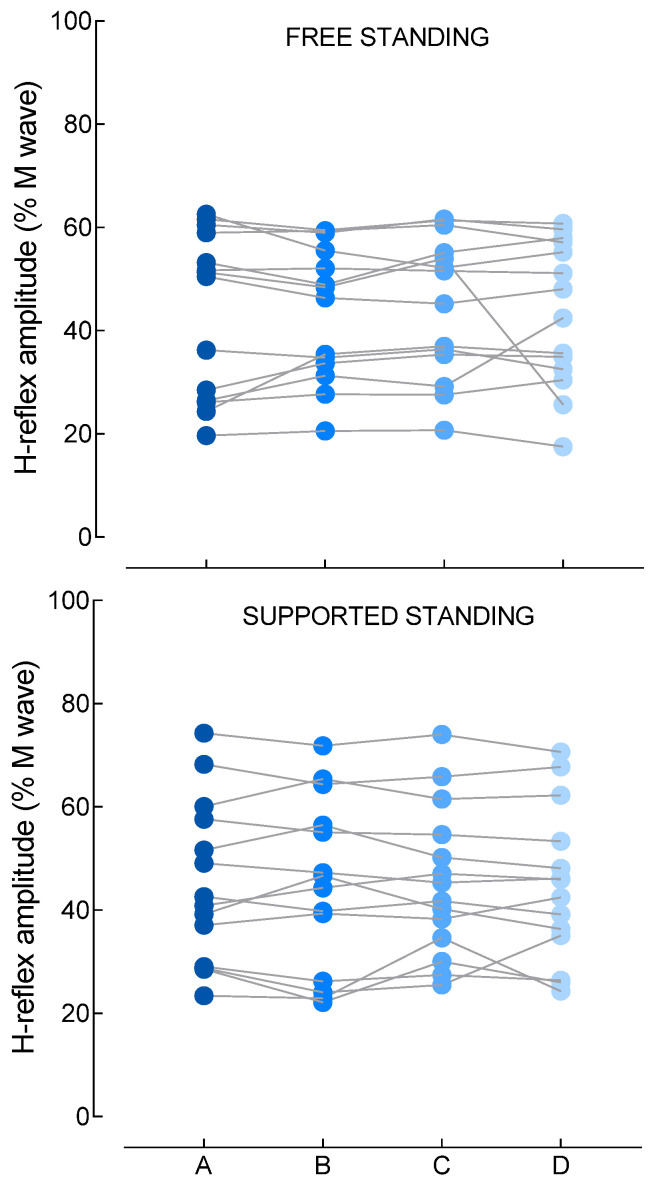
Hoffmann (H) reflex amplitude, expressed as % of the maximal amplitude of the M wave (M_max_) for each condition of optic flow (A, B, C, D). A: no optic flow simulating self-motion; B: alternating optic flow simulating forward and backward self-motion; C: alternating optic flow simulated forward and backward self-motion combined with lateral directions (forward-left or forward-right) and (backward-left or backward-right). D: similar to C with the addition of clockwise and counterclockwise rotations in the visual field. Each point represents one participant.

The main effect of conditions on H-reflex amplitude was not significant [F(3,13) = 0.2, *p* = 0.76, η^2^ = 0.016], with mean amplitude remaining stable across the experimental conditions [A: 43.7 (15.9)% M_max_; B: 43.7 (12.9)% M_max_; C: 44.8 (13.7)% M_max_; D: 43.5 (14.1)% M_max_] (Figure 3). The amplitude of the preceding M wave, used to verify the stability of peripheral stimulation, did not differ across conditions [F(3,13) = 1.1, *p* = 0.37, η^2^ = 0.076], with values ranging from 6.9 to 7.2% M_max_ (Table 1).

Background muscle activity preceding the peripheral nerve stimulation likewise showed minimal sensitivity to the optic flow manipulations. SOL [F(3,13) = 1.5, *p* = 0.25, η^2^ = 0.101] and TA EMG [F(3,13) = 0.95, *p* = 0.42, η^2^ = 0.068] remained unchanged across conditions (Table 1). GM EMG showed a significant effect of conditions [F(3,13) = 6.5, *p* = 0.002, η^2^ = 0.334], although post hoc inspection did not identify any specific differences between conditions (Table 1). This may indicate a lack of clear in-between condition effects or an underpowered analysis for this muscle.

The CoP position just preceding the peripheral nerve stimulation showed no significant effect of the conditions [F(3.13) = 1.2, *p* = 0.33, η^2^ = 0.083], indicating that the mean standing posture at the time of the stimulation was quite similar across conditions.

### 3.2. Supported Standing

In the supported-standing situation, where the postural demands of upright stance were minimized, no significant condition effects were observed across H-reflex amplitude, M-wave amplitude, background EMG, or CoP measures (all *p* > 0.05) (Table 1).

### 3.3. Correlation Analysis

Correlation analyses showed that neither CoP displacement nor pre-stimulus EMG activity predicted variations in H-reflex amplitude across any of the four conditions. Pearson’s coefficient correlations were mainly weak and uniformly non-significant (all *p* > 0.05), demonstrating that CoP velocity and background muscle activity did not account for the absence of reflex modulation (Table 2).

## 4. Discussion

Overall, although the optic-flow stimuli produced clear and consistent increases in CoP velocity, these visually driven perturbations did not translate into detectable changes in SOL H-reflex amplitude. The results therefore suggest that, under the present conditions, optic flow simulating self-motion in upright standing operates predominantly through supraspinal sensorimotor integration mechanisms without requiring specific adjustments of reflex gain within the Ia-monosynaptic pathway.

### 4.1. Optic Flow as a Potent Postural Perturbation

The increased CoP velocity observed in this study aligns with classical and contemporary findings demonstrating the powerful influence of optic flow on postural control. Early work using the moving-room paradigm showed that small visual perturbations produce coherent postural responses [3]. Modern VR studies have since replicated and extended these results, demonstrating that visually induced self-motion elicits significant sway even in the absence of actual body movement [2,4,5]. Furthermore, VR studies manipulating optic-flow velocity demonstrate that even low-velocity perturbations destabilize stance and increase CoP excursion [7], reinforcing the idea that visual motion powerfully influences postural control.

Even though optic flow markedly increased sway relative to the static condition (A), the absence of differences among the three optic-flow conditions (B, C, and D) suggests that once the visual environment imposes self-motion cues strong enough to perturb stance, additional increases in visual complexity or directional variability do not produce proportionally greater postural destabilization. This pattern differs from the results of Ahuja et al. who reported a greater instability when velocity of sinusoidal optic flow was increased [7]. The divergent results may rely on the fact that they compared quite slow velocities (from 0.1 to 10 m/s), whereas optic flow in the present study simulated a displacement of 8 m/s. They also reported no evidence of habituation or progressive amplification of sway over time, implying that postural responses may plateau once visual perturbations exceed a threshold of sensory conflict. When visual input becomes sufficiently unreliable, the central nervous system (CNS) rapidly down-weights it, which results in similarly elevated sway across different forms of optic flow stimulation. The comparable postural responses across conditions B, C, and D in the present study therefore indicate a threshold beyond which additional complexity or directionality in optic flow yields no further behavioral impact.

### 4.2. Absence of Reflex Modulation Under Free-Standing Condition

The lack of H-reflex modulation during free standing contrasts with previous studies reporting reduction in H-reflex amplitude under specific contexts involving threat or heightened arousal. For example, Hodgson et al. (2023) found that VR height exposure increased arousal and postural sway while simultaneously decreasing H-reflex amplitude, implicating supraspinal mechanisms sensitive to psychological state and environmental threat [13]. Similarly, Grosprêtre et al. reported a large decrease in SOL H-reflex amplitude during VR-simulated falling, despite minimal changes in muscle activation or kinematics, suggesting that perceived falling activates protective spinal adjustments typically observed during real balance loss [12]. It should be noted, however, that the main methodological approaches used in previous studies and the present one were not strictly similar. The maximal amplitude of the H reflex [12] or an amplitude half of this [13] were used. This should, however, provide quite similar sensitivity of the H reflex to modulation across studies [18]. In addition, the background EMG of the SOL was checked and did not vary across conditions, as observed in the present study. It is therefore unlikely that the differences underscored above explain the divergent results between previous studies and the present one.

The lack of difference in CoP position at the time of the posterior tibial nerve stimulation largely discards a potential effect of sway phase in the H-reflex responses. Accordingly, the lack of change in H-reflex amplitude in our study may reflect the fact that VR may not have induced sufficient emotional or postural threat. Reflex modulation may appear to arise only when visually induced motion conveys imminent danger or loss of balance.

The results align with contemporary frameworks of sensorimotor integration, which posit that the CNS dynamically distributes control across spinal and supraspinal levels depending on task demands. Sensory reweighting, a key mechanism in postural control models [1], allows the CNS to down-weight unreliable visual cues and increase reliance on other sensory sources during visually induced instability. Recent neurophysiological evidence demonstrates that multisensory integration for postural control involved cerebellar, brainstem, and cortical networks. For example, Mildren and Cullen showed that the cerebellar vermis integrates vestibular and proprioceptive inputs to generate adaptive postural commands [22], with cerebellar pathways being able to facilitate spinal excitability under specific conditions [23]. A computational model further highlights the cerebellum’s central role in combining multisensory signals to support stable motor behavior [24]. The absence of reflex modulation suggests that CNS relies on supraspinal corrective processes rather than spinal mechanisms when dealing with non-threatening visual perturbations.

### 4.3. Optic Flow Alone Does Not Involve H-Reflex Modulation

The supported-standing condition was critical in determining whether optic flow alone (i.e., independent of postural control) could modulate spinal excitability. By removing the need for active balance management, supported standing stabilizes proprioceptive and vestibular signals and minimizes the rapid corrections typically required to maintain upright standing [10,19]. Under such conditions, modulation of the H-reflex pathway should only reflect the processing of conflicing sensory information without any destabilizing challenge. In such situations, no change in H reflex was observed, indicating that sensory conflicts induced by optic flow does not induce modulation within the Ia afferent-motoneuron transmission. In agreement, in VR studies involving simulated height or falling, reflex modulation was never attributed solely to visual input but instead include the emotional and anticipatory consequences of the visual environment. This finding reinforces the notion that postural threat, not optic flow as long as it is not strong enough to induce fall, may underly VR-induced reflex modulation.

### 4.4. Implications, Limitations, and Future Directions

This study indicates that optic flow is a potent visual destabilizer but not a modulator of the Ia afferent-motoneurons pathway, unless embedded within contexts that increase threat, arousal, or anticipation of imbalance. Notably, the present study did not directly assess the threat effect of the optic flow, which could have provided further argument on this point. Nonetheless, the present study has implications for how VR could be used in research and rehabilitation. Interventions targeting spinal reflex modulation must incorporate elements that meaningfully challenge or threaten balance, rather than relying on visual motion alone. Future research should explore how emotional context, cognitive load, and environmental factors interact with optic flow to influence spinal modulation. Studies including older adults or clinical populations may also reveal different patterns of visuomotor integration [25]. Importantly, the lack of change in H-reflex amplitude during upright standing may not necessarily indicate a lack of spinal modulation [18]. For example, Baudry and Duchateau (2012) reported an absence of change in H-reflex amplitude in young and older adults while standing with eyes open and closed, whereas Ia presynaptic inhibition increased from eyes open to eyes closed [26]. Investigating specific spinal inhibitory pathways may offer a comprehensive understanding of how visual motion influences spinal motor control.

## 5. Conclusions

In summary, immersive optic flow significantly destabilizes posture but does not induce modulation of the SOL H-reflex amplitude during upright stance. These results suggest, in our experimental conditions, that visually driven postural perturbations, without the presence of threat or heightened postural challenge, operate predominantly through supraspinal sensory integration mechanisms and are insufficient to induce modulation at the spinal level, at least as assessed by the H reflex. This study thus contributes to a more nuanced understanding of how optic flow interacts with spinal and supraspinal components of postural control and underscores the importance of contextual factors in shaping the relation between optic flow and reflex gain.

## Figures and Tables

**Table 1:** M wave preceding the H reflex, Mmax and background EMG activity in the four optic flow conditions for the two experimental sessions (*Free standing* and *Supported standing*).

Condition	A	B	C	D
***Free standing***				
Preceding M wave (% M_max_)	7.1 (1.0)	6.9 (0.7)	7.2 (1.1)	7.1 (0.8)
M_max_ (mV)	3.3 (1.2)	3.3 (1.2)	3.2 (1.2)	3.2 (1.3)
SOL_EMG_ (μV)	8 (5)	9 (4)	8 (4)	8 (4)
GM_EMG_ (μV)	7 (4)	9 (5)	8 (4)	8 (5)
TA_EMG_ (μV)	5 (2)	5 (2)	5 (2)	5 (2)
***Supported standing***				
Preceding M wave (% M_max_)	6.8 (0.8)	7.0 (0.8)	6.9 (0.7)	6.6 (0.7)
M_max_ (mV)	3.3 (1.5)	3.2 (1.4)	3.3 (1.5)	3.6 (0.9)
SOL_EMG_ (μV)	7 (4)	6 (5)	6 (5)	7 (4)
GM_EMG_ (μV)	6 (3)	9 (5)	8 (4)	8 (5)
TA_EMG_ (μV)	4 (1)	4 (1)	4 (1)	4 (1)

M_max_: maximal amplitude of the M wave; Preceding M wave: M wave that preceded the H reflex during stimulation aimed at evoking H reflex. M wave should be comprised between 5 and 10% M_max_; SOL_EMG_, GM_EMG_ and TA_EMG_: rectified average value of EMG within the 100 ms preceding peripheral nerve stimulation for the soleus, gastrocnemius medialis and tibialis anterior, respectively. A: no optic flow simulating self-motion; B: alternating optic flow simulating forward and backward self-motion; C: alternating optic flow simulated forward and backward self-motion combined with lateral directions (forward-left or forward-right) and (backward-left or backward-right). D: similar to C with the addition of clockwise and counterclockwise rotations in the visual field.

**Table 2:** Pearson’s correlation and associated *p* values between H-reflex amplitude and center of pressure (CoP) position in the antero-posterior axis, electromyography (EMG) activity preceding the posterior tibial nerve stimulation for the soleus (SOL_EMG_), and tibialis anterior (TA_EMG_).

	r	*p*
*Condition A*		
CoP position	0.28	0.340
SOL_EMG_ (μV)	0.49	0.192
TA_EMG_ (μV)	−0.16	0.315
*Condition B*		
CoP position	0.43	0.123
SOL_EMG_ (μV)	0.48	0.221
TA_EMG_ (μV)	−0.18	0.544
*Condition C*		
CoP position	0.17	0.558
SOL_EMG_ (μV)	0.40	0.162
TA_EMG_ (μV)	−0.23	0.437
*Condition D*		
CoP position	0.18	0.550
SOL_EMG_ (μV)	0.22	0.453
TA_EMG_ (μV)	0.18	0.547

A: no optic flow simulating self-motion; B: alternating optic flow simulating forward and backward self-motion; C: alternating optic flow simulated forward and backward self-motion combined with lateral directions (forward-left or forward-right) and (backward-left or backward-right). D: similar to C with the addition of clockwise and counterclockwise rotations in the visual field.

## Data Availability

The original contributions presented in the study are included in the article, further inquiries can be directed to the corresponding author.
